# Seed bank persistence of a South American cordgrass in invaded northern Atlantic and Pacific Coast estuaries

**DOI:** 10.1093/aobpla/plab014

**Published:** 2021-04-08

**Authors:** Ahmed M Abbas, Andrea J Pickart, Laurel M Goldsmith, Desiree N Davenport, Britney Newby, Adolfo F Muñoz-Rodríguez, Brenda J Grewell, Jesús M Castillo

**Affiliations:** 1 Department of Biology, College of Science, King Khalid University 61413, Abha, Saudi Arabia; 2 Department of Botany and Microbiology, Faculty of Science, South Valley University, 83523, Qena, Egypt; 3 U.S. Fish and Wildlife Service, 6800 Lanphere Rd. Arcata, CA 95521, USA; 4 Departamento de Ciencias Integradas, Fuerzas Armadas Ave., Campus El Carmen, Universidad de Huelva, 21071, Huelva, Spain; 5 USDA-Agricultural Research Service, Invasive Species and Pollinator Health Research Unit, Department of Plant Sciences MS-4, University of California, Davis, CA 95616, USA; 6 Departamento de Biología Vegetal y Ecología, Facultad de Biología, Universidad de Sevilla, Apartado 1095, 41080, Sevilla, Spain

**Keywords:** Climate change, germination, invasive species, salt marsh, seed dormancy, *Spartina densiflora*

## Abstract

Invasive alien plant species impart considerable impacts that contribute to the decline of biodiversity worldwide. The ability of an invasive species to overcome barriers to establish and spread in new environments, and the long-term effects of plant invasions supporting their persistence are keys to invasion success. The capacity of introduced species to form soil seed banks can contribute to their invasiveness, yet few studies of invaders have addressed seed bank dynamics. Improved knowledge of this recruitment process can improve conservation management. We studied temporal and spatial changes in soil seed bank characteristics of the cordgrass *Spartina densiflora* from two continental invaded ranges. In the Odiel Marshes (Southwest Iberian Peninsula), *S. densiflora* formed transient seed banks (<1 year). At Humboldt Bay Estuary (California), viable seeds persisted for at least 4 years though the germination percentage fell abruptly after the first year from 29 % to less than 5 % of remaining viable seeds. Total soil seed bank density increased with *S. densiflora* above-ground cover in both estuaries, pointing to the transient component of the seed bank as a critical component of vegetation dynamics during *S. densiflora* invasion. Even so, seed densities as high as c. 750 seeds m^-2^ in Odiel Marshes and c. 12 400 seeds m^-2^ in Humboldt Bay were recorded in some plots without fruiting *S. densiflora* plants. *S. densiflora* spikelet (dispersal unit) density was more than double close to the sediment surface than deeper within soil. Our study shows the importance of evaluating seed banks during the design of invasive species management since seed bank persistence may vary among invaded sites, and can affect the timing and duration required for desired management outcomes.

## Introduction

Invasions by alien plants are a major element of current global change that significantly impact plant community and ecosystem dynamics, and contribute to the worldwide decline of biodiversity (Pyšek and Richardson 2010). The ability of an invasive species to overcome barriers to establish and spread in new environments, and the long-term effects of plant invasions supporting their persistence are keys to invasion success (Gioria *et al.* 2014). The capacity of introduced species to form soil seed banks can contribute to their invasiveness and expansion, yet few studies of invaders have addressed the role of seed banks in the long-term persistence of naturalized populations (Richardson and Kluge 2008; Gioria *et al*. 2012, 2014). Seeds can persist through periods with unfavourable conditions, reducing ecological risks posed by environmental variability (i.e. bet-hedging; Venable and Brown 1988). This ability is especially useful in environments where opportunities for seed germination are infrequent or unpredictable (Harper 1977). Management of invasive species that maintain persistent seed banks can be challenging and often requires long-term monitoring and control efforts to deplete seed banks for overall-success (Panetta and Timmins 2004; Richardson and Kluge 2008). Improved knowledge of seed bank longevity as a recruitment process that supports persistence of invasive alien plants can provide a foundation for conservation management and ecological restoration to mitigate the undesirable impacts of plant invasions on biological diversity (Grewell *et al.* 2019).

 The establishment of transient seed banks in which seeds live in or on the soil for <1 year, or persistent seed banks whose seeds live for >1 year (Thompson and Grime 1979), is determined by environmental factors affecting seed germination and dormancy such as radiation level, temperature and water availability (Walck 2005; Gillard *et al.* 2017; Peng *et al.* 2018). Some plant species can establish both transient and persistent seed banks depending on seed types, seed traits and the effects of environmental factors (Mandák and Pyšek 2001; Cao *et al.* 2012). For example, light requirements, slow germination and the detection of low diurnally fluctuating temperatures enhance soil seed bank persistence by limiting germination (Saatkamp *et al.* 2011). In this context, invasive species may form persistent soil seed banks that promote primary and secondary invasions (Gioria *et al.* 2014; Gioria and Pyšek 2016).

Invasive species usually germinate from persistent seed banks after disturbances that alter environmental conditions such as light and temperature (Presotto *et al.* 2020). Seeds can be resistant to environmental stresses (i.e. desiccation, temperature changes) through dormancy and arrested development if they are retained in persistent soil seed banks (Thompson *et al.* 1997; Long *et al.* 2015). Increased knowledge of seed persistence in soil seed banks can improve modelling efforts to predict the risk of invasive species spread (Guisan and Thuiller 2005).

Coastal salt marshes are good model ecosystems to study seed banks characteristics of invasive species since they are among the most heavily invaded ecosystems in the world (Grosholz 2002). The importance of soil seed banks in the recruitment and persistence of wetland plant communities has long been recognized (i.e. van der Valk and Davis 1978; Leck and Simpson 1987). Halophyte species are known to develop either transient or persistent seed banks when conditions for seed germination are not favorable (Ungar 1995; Polo-Ávila *et al.* 2019). Halophytes and other plant species colonizing high*-*stress arid, saline or floodplain habitats often produce small seeds, establish transient seed banks and have high and fast germination rates that are an adaptive advantage for recruitment during temporarily favourable conditions (Parsons 2012). In contrast, other plant species that persist in highly dynamic or ephemeral environments (e.g. seasonal wetlands; high elevation zones of tidal salt marshes) form persistent seed banks that provide ecological resilience to spatial or temporal variability in disturbances, climate and other changing environmental conditions (Deil 2005; Ooi 2012; Polo-Ávila *et al.* 2019). This variation in seed bank longevity points to the need understanding of seed bank dynamics across habitats for species of conservation concern.

We studied *in situ* seed banks of the austral cordgrass *Spartina densiflora* (Poaceae), native to southern South America. This cordgrass has invaded estuarine salt marshes in Europe’s southwest Iberian Peninsula, northwest Africa and the Pacific Coast of North America from San Francisco Estuary north to Vancouver Island, British Columbia (Bortolus 2006; Castillo *et al.* 2014). *S. densiflora* primarily relies on seeds for reproduction and rarely produces vegetative propagules in the form of extravaginal tillers from the nodes of senescent ramets (Nieva *et al.* 2001b). Native and naturalized populations of *S. densiflora* have low detectable genetic variation (Ayres *et al.* 2008; Castillo *et al.* 2018). In fact, the vast majority of the abundant phenotypic differences recorded in its invasive range are due to phenotypic plasticity rather than local adaption (Castillo *et al.* 2014). Naturalized *S. densiflora* populations in Humboldt Bay, California (Kittelson and Boyd 1997) and in the Iberian Peninsula (Castillo *et al.* 2010) show high fruit (caryopses, simple one-seeded fruit of grasses) set with a high viability rate (Infante-Izquierdo *et al.* 2021). Germination of this invasive species can occur at suboptimal salinity conditions, though germination decreases with increasing salinity (Infante-Izquierdo *et al.* 2019). To date, no other studies have analysed the characteristics of *S. densiflora* seed banks in either the native or the invaded range of the species.

Here we report two complementary studies, one conducted in the European invaded range, and the other in the North American invaded range. The principal objective of each case study was to investigate characteristics of the seed bank in invaded tidal wetlands that can influence invasiveness and persistence of *S. densiflora*. When introduced to new environments, species are thought to have traits that enable tolerance to habitat variation and invasion success (Pyšek *et al.* 2015). Differences in environmental filters among invaded sites can drive trait divergence in exotic species, and insight into variation in traits that promote persistence can inform whether management approaches can be generally applied across invasion sites. Therefore, our second objective was to compare empirical results from the two case studies to gain insights into potential differences in seed bank characteristics of invasive *S. densiflora* between two disjunct naturalized intercontinental ranges. To accomplish these aims, we studied temporal and spatial changes in seed bank characteristics of invasive *S. densiflora* in estuaries from two geographical ranges with contrasting environmental conditions. We evaluated the potential persistence of *S. densiflora* seed bank (transient or persistent), and assessed interannual changes in seed bank size by sampling just before the onset of annual seed dispersal to examine the more persistent component of the seed bank. Finally, we investigated spatial variation in seed bank characteristics among sites within each estuary, variation in seed bank size with soil depth, and temporal dynamics of seed bank depletion. We hypothesized that seed bank size would have higher seed densities at locations with higher above-ground *S. densiflora* cover, due to higher fruit set at these sites. We predicted that *S. densiflora* seed density would be highest near soil surfaces than at depth in the soil. Additionally, we expected to find marked interannual differences in seed bank size that would be correlated with the high interannual variability of Mediterranean climate.

Our results will contribute to knowledge gaps on the regeneration seed bank niche of multi-continental invasions of a wide spread coastal cordgrass in a climate change context. These results can be applied to improve ecological niche models, invasive species risk assessments and management strategies to reverse negative impacts of the biological invasions for conservation of coastal wetland ecosystems.

## Methods

### Study sites

This research reports results from complementary case studies of *S. densiflora*-invaded estuaries in Andalusia, Spain and California, USA. The study in Spain was carried out in the estuary of the Tinto and Odiel rivers that flow into the Gulf of Cadiz, an embayment of the north-east Atlantic Ocean in Europe’s Southwest Iberian Peninsula. The Odiel Marshes experience a hot-dry summer Mediterranean climate (Köppen-Geiger Csa) with some Atlantic influence. Mean annual air temperature range is +17 to +24 °C, average temperature in August is +25 °C, and annual precipitation is 250–850 mm with 75–85 days of rain per year and a 4–5 month dry period from approximately June–September (AEMET 2018; CLIMA 2018). We also studied the seed bank of *S. densiflora* in northwestern California’s Humboldt Bay Estuary (North America). In contrast to Odiel Marshes, Humboldt Bay experiences a warm/cool, dry summer Mediterranean climate (Köppen-Geiger Csb) moderated by cool coastal fog. Mean annual air temperature range is +8 to +15 °C. The warmest month on average is August with a mean temperature of +14 °C. Mean annual rainfall is 1027 mm with 76 days of rain per year and a 2-month dry, but foggy period during July–August (US National Weather Service 2020).

**Figure 1.**
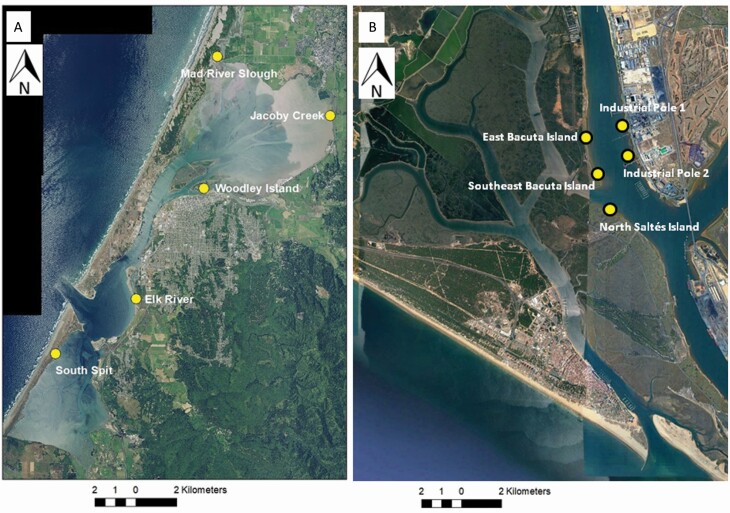
Aerial photographs showing the location of sampled points for *Spartina densiflora* seed banks in (A) Humboldt Bay (California, Pacific Coast of North America) and (B) Odiel Marshes (Gulf of Cádiz, Andalusia, Atlantic Coast of Southwest Iberian Peninsula). Sources and years of aerial photographs: (A) USDA Farm Services Agency, 2020; (B) Instituto Cartográfico de Andalucía, 2000.

The Odiel Marshes are among the most extensive and diverse salt marshes in the Iberian Peninsula, with c. 7160 ha protected as a Natural Park and as a Biosphere Reserve. Five study locations with invasive populations of *S. densiflora* were established in the Odiel Marshes: (1) East Bacuta Island (37°13′21ʺN, 6°57′39ʺW); (2) Southeast Bacuta Island (37°12′56ʺN, 6°57′34ʺW); (3) North Saltés Island (37°12′38ʺN, 6°57′19ʺ W); (4) Industrial Pole 1 (37°13′33ʺN, 6°57′06ʺW); and (5) Industrial Pole 2 (37°13′11ʺN, 6°56′54ʺW) (Fig. 1). Each location includes both low and middle intertidal elevation salt marshes. By definition, low marshes are between Mean High Water Neap (MHWN; c. +2.44 m Spanish Hydrographic Zero (SHZ)) and Mean High Water (MHW; c. +2.91 m SHZ) (Long and Mason 1983). Low marshes support low vegetation cover, with *Sarcocornia perennis* subsp. *perennis* as the dominant plant species, growing in association with isolated tussocks of invasive *S. densiflora* (<20 % cover) and native *Spartina maritima* (<10 %). Middle marshes are from MHW to Mean High Water Spring (MHWS; c. +3.37 m SHZ), and are dominated by a halophyte complex including *Atriplex portulacoides*, *S. densiflora* (>20 % cover) and the hybrid *Sarcocornia perennis × fruticosa* (see **Supporting Information— Fig. S1**).

Humboldt Bay Estuary underwent extensive diking in the early 1900s, and as a result, 90 % of its salt marshes were converted to agricultural lands. The estuary currently has approximately 365 ha of salt marshes. Five extant salt marsh locations invaded by *S. densiflora* were selected as study sites that include island and mainland sites representing geographic variation within the estuary: (1) Mad River Slough (40º52′N, −124º09′W); (2) Jacoby Creek (40º50′N, −124º 5′ W); (3) Woodley Island (40º48′N, −124º09′W); (4) Elk River (40º45′N, −124º11′W); and (5) South Spit (40º44′N, −124º14′W) (Fig. 1). Each study site included two elevation ranges known locally as ‘low marshes’ and ‘high marshes.’ ‘Low marshes’ in Humboldt Bay are those between MHW (c. +1.7 m Mean Lower Low Water (MLLW)) and MHWS (c. +2.2 m MLLW), which corresponds to middle marshes in the Odiel Marshes. Invasive *S. densiflora* (c. 60 % cover) dominated low marshes, in association with native *Sarcocornia pacifica* (syn. *Salicornia pacifica*; c. 25 %) and *Jaumea carnosa* (c. 20 %). High marshes (between MHWS (c. +2.2 m MLLW) and the Highest Astronomical Tide (HAT; c. +2.5 m MLLW) were mainly colonized by *J. carnosa* (c. 30 %), *Distichlis spicata* (c. 20 %) and *S. pacifica* (c. 20 %) (see Supporting Information—Fig. S1).

### Soil seed bank sampling

Seed bank sampling was performed to determine the density of *S. densiflora* seeds within soil seed banks, and the proportions of quiescent and dormant viable seeds and senescent non-viable seeds in the primary intertidal habitats occupied by *S. densiflora* in both estuaries. With this purpose, *S. densiflora* soil seed bank samples were randomly collected during low tides within two intertidal zone habitats using the same collection method at five marsh locations within each estuary (Fig. 1). Soil seed bank cores were taken using 4-cm-deep soil cores with a surface area of 10 × 10 cm. The 4 cm sampling depth was chosen to detect recently dispersed seeds, because buried *S. densiflora* seedlings only emerge from shallow soil layers (Abbas *et al.* 2020). Seed density in salt marshes decreases sharply with sediment depth (Coteff and Van Auken 2006). *Spartina densiflora* disperses its spikelets (caryopses surrounded by bracts) during fall-winter (Nieva *et al.* 1996). Soil seed bank sampling was performed just before spikelet dispersion in early September 2009 (3 initial sites) and 2010 (five locations) at Odiel Marshes, and in August 2010 at Humboldt Bay. To quantify the abundance *S. densiflora* in standing vegetation within study plots, an ocular estimate of relative cover was recorded to the nearest 5 % for 1 × 1 m quadrats centred within each seed bank plot in both Odiel and Humboldt Bay Marshes.

### Odiel Marshes Case Study: seedling emergence assay

To determine the size and viability of the invasive *S. densiflora* seed bank in the Odiel Marshes, 10 replicate samples per each of two occupied intertidal zones were collected at 3 sites in 2009 (3 sites × 2 habitats × 10 reps; *n* = 60 cores). In 2010, the number of sampled locations was increased to five including the addition of sites Industrial Pole 1 and 2 (5 sites × 2 habitats × 10 replicates; *n* = 100 cores). Each soil core sample was divided at two depth intervals (0–2 and 2–4 cm deep), and subjected to a seed bank emergence assay and seed viability assessment (Nicol *et al.* 2003). This method provides an accurate measure of viable seeds and the ability to assess the relationship between seed bank composition and recruitment conditions in wetland soil (Poiani *et al.* 1988; Gross 1990). The experiment was initiated just after sample collection in 2009 and 2010, and carried out in the Greenhouse Facility of the University of Seville. Average daily air temperature during the seedling emergence assay was +21.5 ± 1.5 °C (+12 to +38 °C range). Mean daily relative humidity was 68 ± 3 % (28 to 91 % range). Each soil sample from each depth treatment was homogenized and potted separately in different pots with a maximum depth of 3 cm in well-drained plastic containers before exposing them to germination to ensure seeds were not too deep in the sediments for potential seedlings emergence. Containers were arranged in a complete randomized design and covered with strips of cloth at the bottom to prevent the loss of soil and seeds. Samples were watered daily from above with freshwater (0.5 ppt salinity) in order to maximize germination (Nieva *et al.* 2001a). Seedling emergence was monitored for 8 months when emergence from experimental seed banks had ceased. Seedlings were allowed to grow until they were identified and counted. This method allowed for an estimate of the viable portion of the seed bank and enabled the identification of the emergent flora to species level, although it may fail to detect dormant seeds whose germination requirements would not be met under greenhouse conditions (Gioria *et al.* 2014). Experimental seed banks that produced emergent seedlings were considered to be in a quiescent state in the field since they germinated when transferred to suitable conditions in the greenhouse (Baskin and Baskin 1985). At termination of the emergence assay, all soil was sieved to obtain any remaining *S. densiflora* caryopses in the form of spikelets. Then, viability of all ungerminated seeds was checked using the tetrazolium test. Floral bracts were removed from each spikelet before the caryopses were cut longitudinally and soaked in a 1 % tetrazolium chloride solution at +25 °C for 24 h. Seeds were then examined under a dissecting microscope, and were considered viable when more than 50 % of the embryo was stained red (Baskin and Baskin 2004); otherwise seeds were considered senescent (Leist *et al*. 2003).

### Humboldt Bay Case Study: temporal and spatial variation in seed banks

At Humboldt Bay, sampling was performed to determine the size and viability of the seed bank at study sites. In 2010, following methods previously described, we collected 20 replicate soil core samples at five study sites to characterize the *S. densiflora* soil seed bank in low and high elevation marshes (5 sites × 2 habitats, *n* = 200 cores).

A 4-year field study was also implemented to evaluate temporal variation in seed bank longevity within and among sites under natural environmental conditions in low elevation marshes, where density of invasive *S. densiflora* was maximal. We randomly established 20 monitoring plots (1 m^2^) in each of five study marshes. Within each plot, any plants present within the centre 30 × 30 cm of the plot (and a buffer of 10 cm) were clipped to ground level. A mesh screen (<1-mm) that covered the subplot and its edges was inserted in a shallow slit on each side of the subplot. Landscape staples were used to anchor all sides of the screen, thus ensuring that no new seed could reach the sediment surface. The reduction of radiation due to the presence of the covering mesh screen should not alter the germination of *S. densiflora* because it shows similar germination percentages in light and darkness (Nieva *et al.* 2001a). Plots were visited once annually during winter in each of 3 years (2011–13) following the initial sampling in 2010. Before the onset of annual, seasonal seedling emergence in spring, the screen was removed, and soil sample replicates were extracted from the 30 × 30 cm screened area within each of the 20 plots (2011–13: 5 sites × 1 habitat, *n* = 100 cores × 3 years = 300 total cores). Immediately after field collection, soil cores were sieved to obtain *S. densiflora* caryopses in the form of spikelets. Following Infante-Izquierdo *et al.* (2019), spikelet germinability was tested in freshwater conditions using distilled water in Petri dishes (9 cm diameter) under 12 h/12 h photoperiod conditions and a +20–25 °C air temperature range. Seeds were considered germinated when radicle emergence was visible. Viability of remaining ungerminated seeds was assessed using the tetrazolium test previously described. Seed density was calculated as the number of *S. densiflora* seeds per square metre. Viable and senescent seeds were each expressed as the percentage of the total number of seeds per sample.

### Statistical analysis

All statistical analyses were carried out using SPSS release 15.0 (SPSS Inc.). Deviations were calculated as standard errors of the mean (SEM). A significance level (α) of 0.05 was applied for every analysis. Data were tested for normality with the Kolmogorov–Smirnov test and for homogeneity of variance with the Levene test. Additionally, in independent analyses for each estuary, Spearman correlation (ρ) coefficient was used to examine relationships between the seed bank density and standing *S. densiflora* cover. To determine the size and viability of the seed bank of *S. densiflora* in the Odiel Marshes, non-parametric Kruskal–Wallis ANOVA (H) and Mann–Whitney *U*-test as *post hoc* analysis were applied to compare spikelet densities quantified from soil core collections among locations. Wilcoxon test (Z) was used to compare spikelet densities between middle and high marshes, sediments depths and sampling years at the Odiel Marshes. For the Humboldt Bay case study, Kruskal–Wallis ANOVA (H) and Mann–Whitney *U*-test as *post hoc* analysis were applied to compare spikelet density among locations, and also to compare the percentage of senescent seeds among locations and among years for each location. Additionally, spikelet density was compared between low and high marshes using Wilcoxon test.

## Results

### Seedling emergence assay: Odiel Marshes

**Figure 2.**
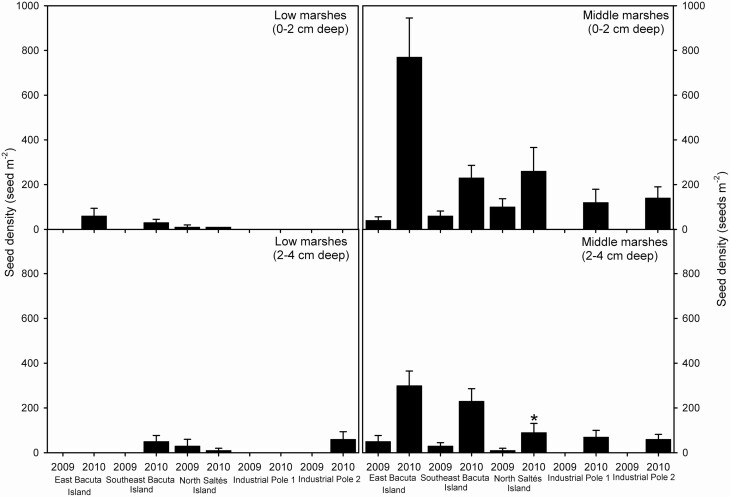
Senescent non-viable seed densities (seed m^-2^) of invasive *Spartina densiflora* prior to seed dispersal at two sediment depths (0–2 cm, 2–4 cm deep) in low and middle marshes at five locations in the Odiel Marshes, Southwest Iberian Peninsula in 2009 and 2010. Data are means (*n* = 10 soil seed bank samples). *One seed germinated from this sample.

In the Odiel Marshes, only one *S. densiflora* seedling emerged during the assay period in the greenhouse; this seedling emerged from sediments collected in Saltés Norte middle marsh between 2 and 4 cm deep in 2010 (see **Supporting Information— Table S2**). At the end of the 8 month assay, all ungerminated *S. densiflora* seeds were senescent/non-viable (Fig. 2).

**Figure 3.**
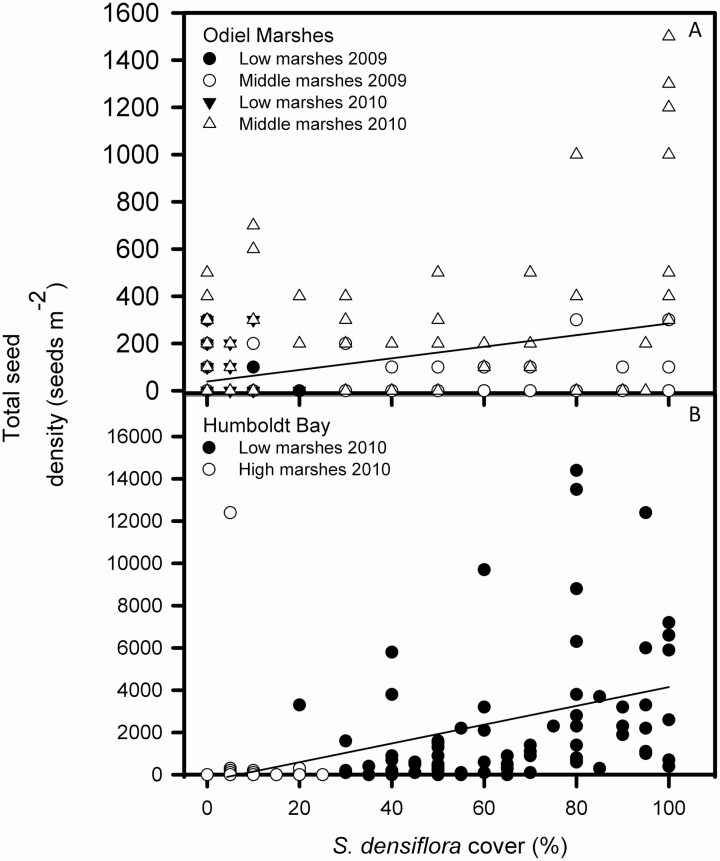
Relationships between *Spartina densiflora* seed bank size (total seed (dead + viable) m^-2^) and standing relative cover (%) in (A) Odiel Marshes (Southwest Iberian Peninsula) and (B) Humboldt Bay (California). Low marshes in Humboldt Bay correspond to middle marshes in the Odiel Marshes. Regression equations: (A) y = 39.101 + 2.455 x (ρ = 0.337, *P* < 0.0001, n = 320); (B) y = -291.195 + 44.385 x (ρ = 0.821, *P* < 0.0001, n = 194).

Total spikelet density showed no significant differences for the five sampled locations in the Odiel Marshes (Kruskal–Wallis, χ ^2^ = 4.627, d.f. = 4, *P* > 0.05). Comparing habitats along the intertidal gradient, total spikelet density was more than 10 times higher in middle than in low marshes (Wilcoxon test, Z = -3.352, *P* < 0.001) (Fig. 2). Total spikelet density increased together with *S. densiflora* cover (Spearman correlation, ρ = +0.337, *P* < 0.0001, *n* = 320; Fig. 3). In general, comparing sediment depths, total spikelet density was more than double between 0 and 2 cm deep (108 ± 19 seeds m^-2^) than between 2 and 4 cm deep (62 ± 10 seeds m^-2^) (Wilcoxon test, Z = -2.368, *P* < 0.05); this difference was mainly due to the data obtained in middle marshes rather than in low marshes (Fig. 2). Total spikelet density was much lower in 2009 (28 ± 6 seeds m^-2^) than in 2010 (119 ± 16 seeds m^-2^) (Wilcoxon test, Z = -3.174, *P* < 0.005) (Fig. 2). The percentage of senescent seeds prior to spikelet dispersion after the subsequent flowering was 100 % for every location, except at Saltés Norte in 2010 (53 %) where just one *S. densiflora* seed germinated.

### Temporal and spatial variation in seed banks: Humboldt Bay

**Figure 4.**
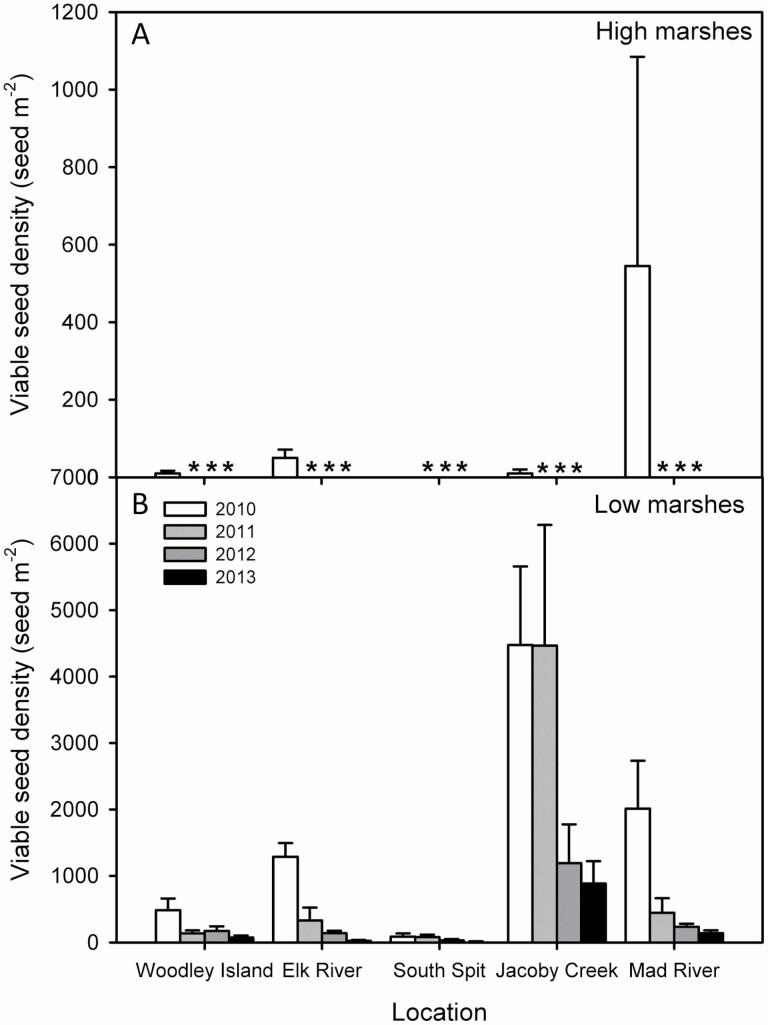
Viable seed density (seed m^-2^) in the seed banks of invasive *Spartina densiflora* prior to seed dispersal (A) in high (2010) and (B) in low marshes from 2010 to 2014, at five locations in Humboldt Bay Estuary, California. Data are means ± SE (*n* = 20 soil seed bank samples). Low marshes in Humboldt Bay correspond to middle marshes in the Odiel Marshes. *Data not recorded.

At Humboldt Bay, 450 seeds (29 % of viable seeds) germinated from the seed bank in the first sampling, dropping markedly to 32 germinated seeds (4 % of viable seeds) in the second sampling year, and to 8 and 3 germinated seeds in the third and fourth years during depletion of the seed bank via seedling emergence over time at the screened plots. Viable (quiescent/germinated and viable dormant) seed percentage decreased from 76 to 50 % of total spikelets from the first to the fourth year of monitoring (Table 1). Average viable seed density in *S. densiflora* remnant seed bank was 900 ± 451 seeds m^-2^ in Humboldt Bay; with maximum of 227 viable seeds per sample. Following the exhaustion of the recorded remnant seed bank, viable seeds were recorded at every location in Humboldt Bay until the fourth year, when viable seed density varied between 10 ± 7 seed m^-2^ and 886 ± 336 seed m^-2^ (Fig. 4).

**Table 1.** Seed percentage (%) and number (*n*) of seeds remaining in plots during temporal exhaustion of *Spartina densiflora* seed bank in low salt marshes at five locations in Humboldt Bay (northern California). Plots were covered with a mesh screen to ensure that any potential incoming seed could not reach the sediment surface. Viable seeds include germinated and dormant seeds.

Year	Viable seeds remaining from the first year	Viable seeds compared with total remaining seeds	Germinated seeds in relation to remaining viable seeds
2010	100 %	76 % (*n* = 1541)	29 % (*n* = 450)
2011	56 %	74 % (*n* = 869)	4 % (*n* = 32)
2012	19 %	63 % (*n* = 291)	3 % (*n* = 8)
2013	11 %	50 % (*n* = 174)	2 % (*n* = 3)

Comparing habitats along the intertidal gradient, total spikelet density was more than 10 times higher in low than in high marshes in Humboldt Bay (Wilcoxon test, Z = -7.438, *P* < 0.0001) (Fig. 4). Total spikelet density varied significantly among locations in Humboldt Bay (Kruskal–Wallis test, χ ^2^ = 18.150, g.l. = 4, *P* < 0.001). The remnant seed bank composed of viable seeds was more than 42 times larger at Jacoby Creek in East North Bay than at South Spit in West South Bay (Mann–Whitney *U*-test, *P* < 0.0001) (Fig. 4). Total spikelet density increased together with *S. densiflora* cover in Humboldt Bay (Spearman correlation, ρ = +0.821, *P* < 0.0001, *n* = 194) (Fig. 3).

In Humboldt Bay, the density of viable seeds in low marshes declined steeply after the first year, except in Jacoby Creek and South Spit. In these two locations, similar viable seed densities were recorded for the first two sampled years, declining abruptly in the third year. By the fourth year, viable seed density ranged from 10 ± 7 seed m^-2^ at South Spit to 886 ± 336 seed m^-2^ at Jacoby Creek. The highest decrease in seed density in the fourth year was recorded in Elk River Slough (-98 %) and the lowest in Jacoby Creek (-80 %) (Fig. 4).

**Figure 5.**
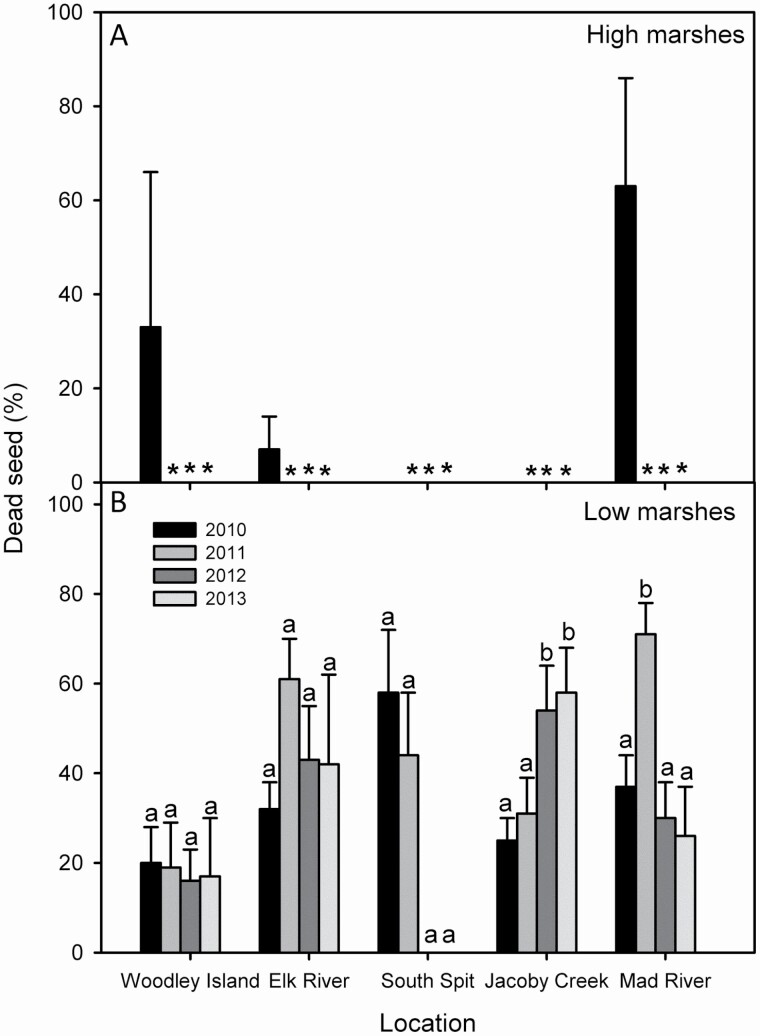
Percentage of senescent non-viable seeds prior to seed dispersal for invasive *Spartina densiflora* seed banks at (A) high and (B) low marshes in Humboldt Bay (California) in 2010, 2011, 2012 and 2013. Data are means ± SE (*n* = 20 soil seed bank samples). Low marshes in Humboldt Bay correspond to middle marshes in the Odiel Marshes (Southwest Iberian Peninsula; see Fig. 3). *Data not recorded. Different letters indicate significant differences among years in the same location (Kruskal–Wallis, *P* < 0.05; Mann–Whitney *U*-test, *P* < 0.05).

The percentage of senescent seeds prior to dispersion after the subsequent flowering changed between c. 20–60 % at different locations in Humboldt Bay (Kruskal–Wallis, χ ^2^ = 9.674, g.l. = 4, *P* < 0.05; Mann–Whitney *U*-test, *P* < 0.05). In Humboldt Bay, the percentage of senescent seeds did not change significantly among years at Woodley Island, Elk River and South Spit (Kruskal–Wallis, *P* > 0.05), increasing after the second year at Jacoby Creek, and being the highest in the second year at Mad River Slough (Kruskal–Wallis, *P* < 0.05; Mann–Whitney *U*-test, *P* < 0.05) (Fig. 5).

## Discussion

Seed bank influences on plant population dynamics are determined by seed production, persistence and turnover (Leck *et al.* 1989). Our reported results from two independent studies reveal high spatial and temporal differences of invasive *S. densiflora* soil seed bank composition, persistence and dynamics between two estuaries in its naturalized intercontinental ranges (Europe and North America). *Spartina densiflora* formed transient seed banks in the Odiel Marshes and short-term persistent seed banks at Humboldt Bay with high inter-population and inter-annual differences. Thus, our results pointed to the transient component of the seed bank as a critical constituent of vegetation dynamics during *S. densiflora* invasion.

The seedling emergence assay suggests the existence of a viable *S. densiflora* transient seed bank at Odiel Marshes. Even though we did not test for seed viability or germination at the beginning of the assay, the seed bank may have been viable initially and seeds were likely able to germinate, since previous works have reported high *S. densiflora* seedling emergence in the first year after seed dispersal (Nieva 1996; Abbas *et al.* 2014). In addition, other observations made by our group in Odiel Marshes also show seed bank transience (data not shown).

Many perennials commonly found in coastal salt marshes do not produce persistent seed banks (Ungar 1991), which may be due to the loss of seeds because tidal abrasion (Hutchings and Russell 1989), short life of seeds with a thin seed coat (Shin-Chin *et al*. 1994) and abiotic environmental extremes beyond the range of tolerance for the seeds of many species (Ungar 1991). Previous studies on *Spartina* species have found transient seed banks for *S. alterniflora* (Hartman 1988; Wang *et al.* 2009; Xiao *et al*. 2009, 2016), *Spartina foliosa* (Hopkins and Parker 1984), *Spartina anglica* (Ungar and Woodell 1993), *Spartina argentinensis* (Feldman *et al.* 2007) and *Spartina maritima* (Polo-Ávila *et al.* 2019). In stark contrast to our results from Odiel Marshes, *S. densiflora* was able to establish a short-term persistent seed bank at Humboldt Bay with viable seeds in soil maintained for at least 4 years. Despite the differences in methods used to explore aspects of seed bank dynamics at each estuary, the observed differences in seed bank longevity are notable. This seed bank persistence observed at Humboldt Bay could confer a strong competitive advantage to *S. densiflora* as an invader, allowing the seed bank to accrue annually and buffer against years of poor environmental conditions, seed production and/or seedling survival. Despite these seed bank differences between our study sites, *S. densiflora* has been able to invade most of the available habitat in large extensive marshes at both Humboldt Bay and Odiel Marshes, where it has established large monospecific stands (Kittelson and Boyd 1997; Nieva *et al.* 2001b). In addition, *S. densiflora* now continues to spread in both study estuaries due to high annual seed production and seedling recruitment. For example, *S. densiflora* is able to produce more than 10 000 caryopses m^−2^ in low marshes and salt pans in the Odiel Marshes (Infante-Izquierdo *et al.* 2021). The establishment of the persistent seed bank of *S. densiflora* would be possible due to the non-deep physiological seed dormancy recorded for many *Spartina* species (Baskin and Baskin 2014). Dormancy is frequent in many halophytes that disperse during the fall season (Baskin and Baskin 2014), promoting germination when favourable establishment conditions occur in spring (Ungar 1978), after the high river outflow in winter that can dislodge seedlings from sediments. Nevertheless, in our study, the germination percentage in Humboldt Bay fell abruptly after the first year from 29 % to less than 5 % of remaining viable seeds. This may indicate that seeds recorded as viable using the tetrazolium test did not retain the ability to germinate due to the loss of seed vigour by seed deterioration that affects many enzymes and organelles with ageing (Enju *et al.* 1993). Aged seeds become more sensitive to stresses during germination and can ultimately be ungerminable due to internal and external factors (Walters *et al.* 2005; Rajjou and Debeaujon 2008). These factors that restrain germination could limit the actual life span of *S. densiflora* seed bank in Humboldt Bay to just 1 year, being residual afterwards.

Previous studies have reported that seed longevity is favoured and seed germination and emergence from the seed bank is reduced by low temperatures (Middleton and McKee 2011; Yan *et al* 2012; Escobar and Cardoso 2015). Moreover, Abbas *et al.* (2014) found germination percentage increased and quiescent and dormant percentages decreased for *S. densiflora* with increasing daily sediment temperatures and daily temperature fluctuations. Thus, seed persistence may be higher in Humboldt Bay than in the Odiel Marshes due to lower air temperatures **[see ****Supporting Information—Fig. S2**, **Table S2****]**. Besides climatic conditions, other environmental factors such as sediment salinity and biotic factors (e.g. seed predation), and even differences in methods used between our case studies, cannot be ruled out as concomitant causes of the differences in the seed bank persistence we recorded between estuaries in different continental ranges of invasive *S. densiflora.*

Besides differences in seed bank characteristics between distant geographical locations, we also recorded spatial differences in seed banks within estuaries. Total spikelet density increased with increases in the relative above-ground cover of *S. densiflora*. For example, at most study sites in both estuaries where relative cover of standing *S. densiflora* was high (>80 %), seed density was also high with >700 seeds m^-2^ in Odiel Marshes, and >11 000 seeds m^-2^ in Humboldt Bay. Thus, differences in the invasion stage produced marked differences on soil seed bank density between habitats along the intertidal gradient and among locations in both studied estuaries. However, we found that some sites with dense *S. densiflora* cover had no seeds or a very low density of *S. densiflora* seeds. Nieva *et al.* (2005) described that flowering can be very low in dense and mature *S. densiflora* populations. This may be explained by a reduction in light intensity where intra-tussock tiller density is high, and this leads to lower production of flowering tillers (Nieva *et al.* 2001b). The high correlation between *S. densiflora* cover and spikelet density in soil seed banks suggests seed bank size of the invader is more dependent on local seed production than on seed dispersal over medium-long distances. In the Odiel Marshes, the highest densities of spikelets in seed banks were recorded in middle intertidal marshes invaded by dense patches of large perennial *S. densiflora* tussocks. Within this middle zone more spikelets are produced than in low marshes (Nieva *et al*. 2001b, 2005), where *S. densiflora* populations are biannual with sparse and small tussocks (Castillo and Figueroa 2007). Even so, seed densities as high as c. 750 seeds m^−2^ in Odiel Marshes and c. 12 400 seeds m^-2^ in Humboldt Bay were recorded in some plots without fruiting *S. densiflora* plants in standing vegetation or in areas with low above ground cover of *S. densiflora*, suggesting seeds were produced elsewhere and immigrated to form seed banks, reflecting a high capacity for hydrochorous dispersal (McDonald 2014).

Results from our seedling emergence assay indicate *S. densiflora* spikelet density was higher close to the sediment surface than deeper in the soil. A steady decrease in the number of seeds with soil depth has often been recorded in wetlands (Leck *et al.* 1989), although the depletion of the seed bank in the upper soil layer is expected to be faster than exhaustion from deeper soil layers due to greater reduction through germination or predation (Dekker 1999). In fact, the only *S. densiflora* seedling recorded in the Odiel Marshes emerged from soil within 2–4 cm of the marsh surface. The higher spikelet density in the upper soil layer may result from direct deposition on the soil surface together with low sediment accretion and disturbance rates (Keddy and Reznicek 1982). In addition, processes ongoing during and after seed rain and incorporation into the soil, such as decomposition and displacement, can result in lower seed accumulation in deeper soil layers (Espinar *et al.* 2005).

The total spikelet density of *S. densiflora* changed abruptly between years in Odiel Marshes. The marked interannual variation we observed is characteristic of transient seed banks that reﬂect the output of the current vegetation rather than that of the vegetation growing at some time in the past (Hutchings and Russell 1989). This could be related to different environmental factors influencing flowering, spikelet production and dispersion such as meteorological conditions and seed predation. For example, rainfall may play a prominent role in regulating seed bank formation in saline communities (McMahon and Ungar 1978).

## Conclusions

The invasive spread of *S. densiflora* is explained primarily by seed dispersal and establishment, and, therefore, understanding the variation in seed production and seed bank dynamics is crucial to successful efforts to mitigate negative impacts of *S. densiflora* invasions. This species rarely produces extravaginal tillers, rhizome fragmentation is scarce and the short rhizomes produced only support very local clonal (vegetative) expansion limited to small distances around its tussocks (Nieva *et al.* 2005). In view of our results, it seems that measures to counter *S. densiflora* invasion will be most effective and eradication should be more economically feasible where the species does not establish a persistent seed bank. In these areas, timing of control efforts is critical, and no additional management measures may be necessary if the physical or chemical elimination of *S. densiflora* plants can be carried out during summer months before the seeds have ripened and dispersal begins. In contrast, where persistent seed banks are formed, managers will need to plan long-term management efforts, adding to the cost of conservation actions. In cases, where a persistent seed bank is present, elimination of new plants emerging from the seed bank should be carried out until the seed bank is exhausted, and before the second year after seedling emergence is observed because the plants first set fruits and produce seeds at this age (Castillo and Figueroa 2007). Additionally, since seed bank composition and size changes markedly among different sites, our results indicate site-specific seed bank studies with assessment of local seed bank persistence are recommended prior to the onset of any management actions to develop effective control strategies tailored to the location. This knowledge will also inform improved planning and allocation of resources needed to accomplish effective management.

## Supporting Information

The following additional information is available in the online version of this article—

**Figure S1**. Photographs of *Spartina densiflora* invasion in low (a, c) and middle-high (b, d) salt marshes in Odiel Marshes (Southwest Iberian Peninsula; a, b) and Humboldt Bay (northern California; c, d).

**Figure S2**. Monthly rainfall (mm; bars) and maximum (triangle) and minimum (circle) air temperature (°C) in (a) Odiel Marshes from January 2008 to December 2010, and in (b) Humboldt Bay from October 2010 to September 2014. Meteorological data for the study periods were obtained from ‘Francisco Montenegro’ meteorological station located near the Odiel Marshes (37º16’ N - 06º57’ W) and from the US National Weather Service meteorological station at Woodley Island, Humboldt Bay (40º81’ N, -124º16’ W).

**Table S2**. Monthly air temperatures during the study period, monthly average estuarine water temperature and salinity in Humboldt Bay (northern California) and Odiel Marshes (SW Iberian Peninsula).

plab014_suppl_Supplementary_Material

## Data Availability

All code and data used in this study are available at Abbas_et_al_20180S1. Figshare. Dataset. https://doi.org/10.6084/m9.figshare.14192684.v1

## Notes

**Populations & Communities. Chief Editor:** Jean Burns
