# Supplementary material for: Seed bank persistence of a South American cordgrass in invaded northern Atlantic and Pacific Coast estuaries
Source: AoB Plants. 2021 Apr 8;13(3):plab014. doi: 10.1093/aobpla/plab014 (PMC8112770; doi:10.1093/aobpla/plab014)
Supplement: plab014_suppl_Supplementary_Material [file plab014_suppl_supplementary_material.pdf]

Supporting Information

Seed bank persistence of a South American cordgrass in invaded northern Atlantic and Pacific Coast estuaries

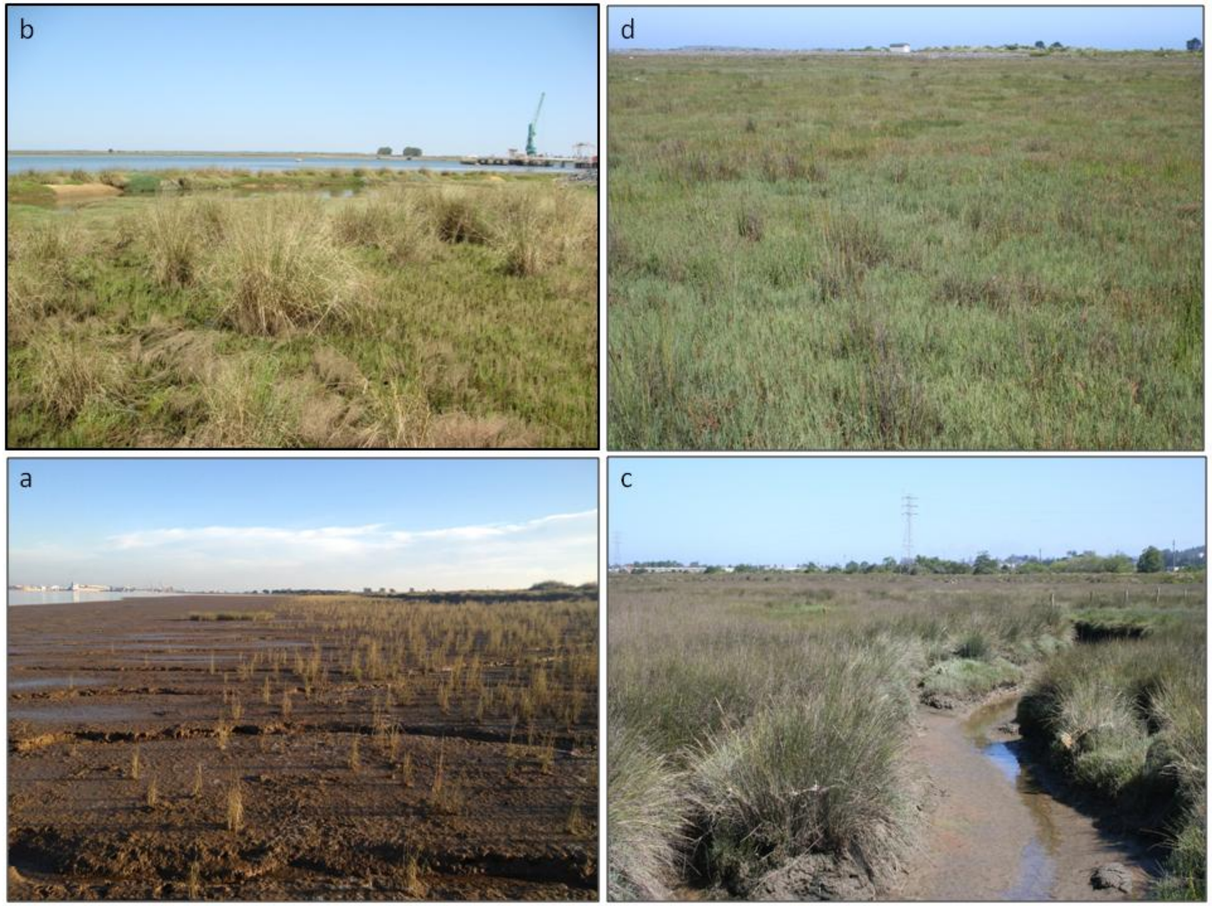

Fig. S1 *Spartina densiflora* invasion in low (a, c) and middle-high (b, d) salt marshes in Odiel Marshes (Southwest Iberian Peninsula; a, b) and Humboldt Bay (northern California; c, d)

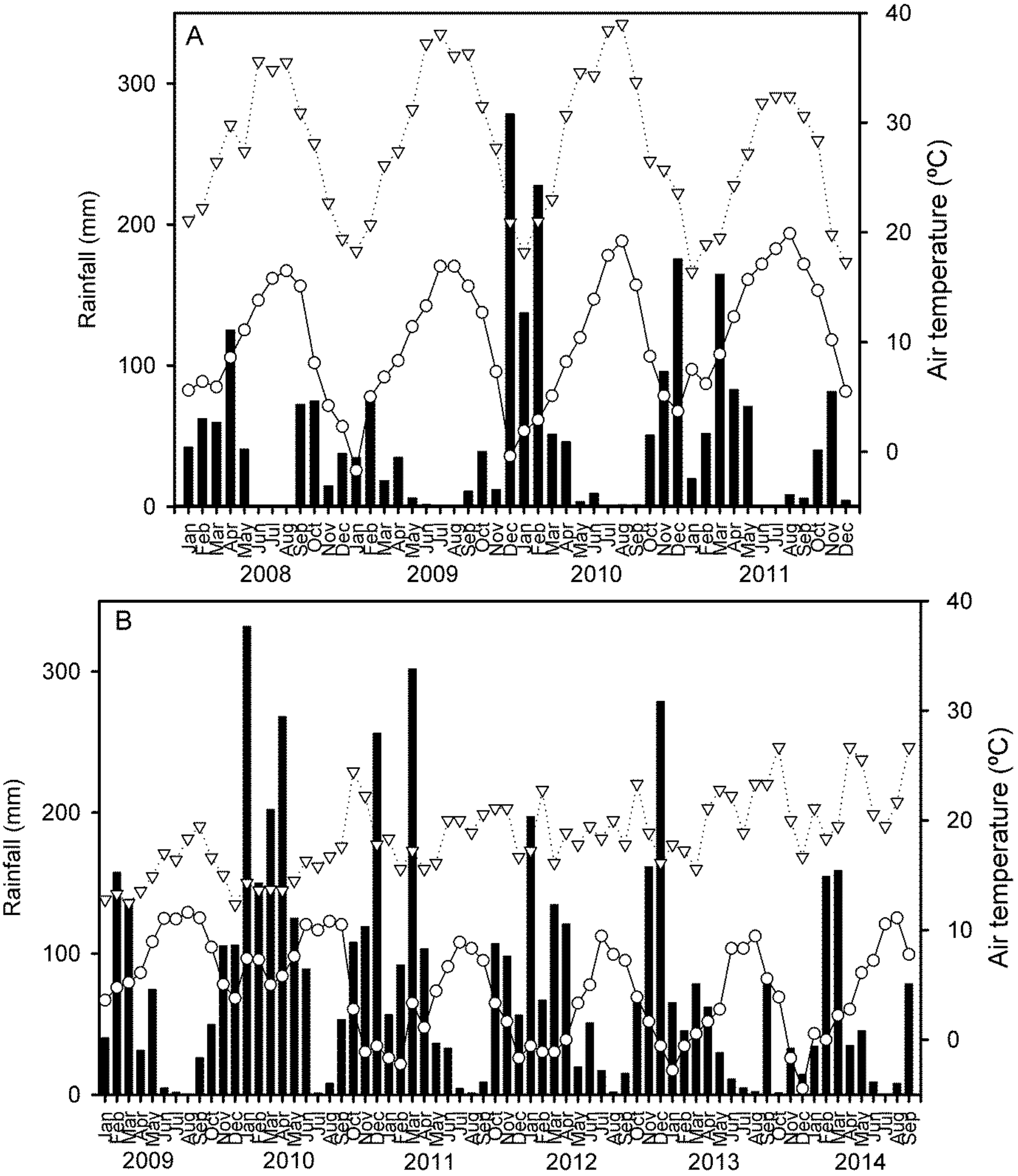

Figure S2. Monthly rainfall (mm; bars) and maximum (triangle) and minimum (circle) air temperature (ºC) in (a) Odiel Marshes from January 2008 to December 2010, and in (b) Humboldt Bay from October 2010 to September 2014. Meteorological data for the study periods were obtained from 'Francisco Montenegro' meteorological station located near the Odiel Marshes (37º16' N - 06º57' W) and from National Weather Service meteorological station at Woodley Island in Humboldt Bay (40º81' N, -124º16' W).

Table 1. Number of emerged seedlings per species from 1000 cm^2 of low and middle salt marshes (10 plots of 10 x 10 cm) at two sediment depths (0-2 cm and 2-4 cm) for five locations in the Odiel Marshes in September 2009 and 2010. Plant taxa: Ap, *Atriplex portulacoides*; Sa, *Salicorniae*; **Sd**, *Spartina densiflora*; Su, *Suaeda* sp.

Location	Habitat	Depth (cm)	# seedlings 2009	# seedlings 2010
East Bacuta Island	Low marsh	0-2	0	0
		2-4	0	0
	Middle marsh	0-2	31 Sa	119 Sa, 1 Ap,
		2-4	2 Sa	43 Sa
Southeast Bacuta Island	Low marsh	0-2	0	0
		2-4	0	0
	Middle marsh	0-2	16 Sa, 1 Ap	82 Sa, 1 Ap,
		2-4	37 Sa, 2 Ap	29 Sa
North Saltés Island	Low marsh	0-2	0	0
		2-4	0	0
	Middle marsh	0-2	106 Sa, 1 Su	104 Sa, 1 Ap,
		2-4	20 Sa	33 Sa, **1 Sd**
Industrial Pole 1	Low marsh	0-2	-	0
		2-4	-	0
	Middle marsh	0-2	-	44 Sa
		2-4	-	82 Sa
Industrial Pole 2	Low marsh	0-2	-	0
		2-4	-	0
	Middle marsh	0-2	-	38 Sa
		2-4	-	44 Sa

Table S2 Monthly air temperatures during the study period, monthly average estuarine water temperature and salinity in Humboldt Bay (northern California) and Odiel Marshes (SW Iberian Peninsula).

Estuaries	Monthly average air temperature (ºC)	Monthly average maximum air temperature (ºC)	Minimum / Maximum air temperatures (ºC)	Monthly temperature fluctuation (ºC)	Monthly water temperature (ºC)	Water salinity (PSS)
Humboldt Bay	11.6 ± 0.3	18.5 ± 0.4	-4.4 / 26.7	13.8 ± 0.7	12.5 ± 1.3[a]	Usually > 27[c]
Odiel Marshes	19.3 ± 1.3	29.2 ± 1.4	-1.7 / 39.0	19.9 ± 0.4	18.7 ± 0.8[b]	18-33[d]

Sources: a, NOAA 2020; b, World Sea Temperature 2020; c, CeNCOOS 2020; d, Sánchez-Rodas *et al*. 2005

CeNCOOS (2020). Central and Northern California Ocean Observing System. Humboldt Bay. https://www.cencoos.org/data-by-location/humboldt-bay/. Accessed 16 July 2020.
NOAA (2020) https://www.ndbc.noaa.gov/ Accessed 10 September 2020
Sánchez-Rodas D, Gomez-Ariza JL, Giraldez I, Velasco A, Morales E (2005) Arsenic speciation in river and estuarine waters from southwest Spain. Sci Total Environ 345: 207-217. https://doi.org/10.1016/j.scitotenv.2004.10.029
World Sea Temperature (2020) https://www.seatemperature.org/. Accessed 10 September 2020
